# Ultrasonic-Assisted Glycosylation with Glucose on the Functional and Structural Properties of Fish Gelatin

**DOI:** 10.3390/gels9020119

**Published:** 2023-02-01

**Authors:** Wenwen Guo, Keying Ding, Kaiyuan Su, Wanyi Sun, Shengnan Zhan, Qiaoming Lou, Tao Huang

**Affiliations:** 1 College of Food and Pharmaceutical Sciences, Ningbo University, Ningbo 315800, China; 2 Key Laboratory of Animal Protein Food Deep Processing Technology of Zhejiang Province, Ningbo University, Ningbo 315800, China; 3 Zhejiang-Malaysia Joint Research Laboratory for Agricultural Product Processing and Nutrition, Ningbo 315800, China

**Keywords:** ultrasound-assisted glycosylation, fish gelatin, functional and structural properties

## Abstract

The effects of ultrasound-assisted glycosylation (UG) with glucose (GLU) on the emulsifying properties, foaming properties, gelling properties, and structural properties of fish gelatin (FG) were investigated. It was shown that UG with high power and a long duration facilitated the Maillard reaction through the reduction of the free amino acid contents. UG significantly improved the emulsifying ability index and foaming capacity of FG whilst decreasing the gel strength. Rheological analysis showed that UG modification prolonged the gelling time by hindering the triple-helix formation and decreasing the apparent viscosity of the gelatin solution. Structural analysis showed that UG treatment changed the secondary structure of the gelatin molecule by the formation of Millard reaction products (MRPs). Moreover, the UG treatment generally decreased the bound water contents of the gelatin gels with an increase in free water.

## 1. Introduction

Gelatin is a collagen hydrolysis product developed through thermal denaturation or physical and chemical degradation [1]. Gelatin has been widely applied in the food, pharmaceutical, and cosmetic industries owing to its gelling, emulsifying, thickening, and stability properties [2]. Nowadays, in the gelatin market, 98.5% of commercial gelatin is derived from mammals. Meanwhile, owing to African swine fever and foot-and-mouth disease, consumers still question the safety of mammalian gelatin [3]. Moreover, plenty of social–cultural and religious beliefs have also restricted the development and usage of mammalian gelatin. Thus, it is very interesting to develop new gelatin sources that can replace mammalian gelatin [2,4]. Fish gelatin (FG), a collagen product, is extracted from fish processing by-products (e.g., fish scales, skins, bones, and swimming bladder). FG has multi-functional physical properties that are similar to those of mammalian gelatin, and thus, FG is considered a potentially excellent mammalian gelatin substitute. However, compared to mammalian gelatin, its surface and gelling properties are still very low [1,5].

At present, lots of modification methods have been reported to enhance the gelling properties of FG: enzymatic modification (transglutaminase, Protamex) [2,6], chemical modifications (phosphorylation, aldehyde, glycosylation) [4,7], and physical modifications (ultrasound, microwave, ultraviolet irradiation, and high pressure) [8,9,10]. Among all the modifications, glycosylation modification of FG is still less reported. The free amino groups (mainly ε-amino) in the side chains of protein amino acids could be covalently crosslinked with the reductive carbonyl end of sugars through a glycosylation reaction [3]. Our previous reports have reported that glycosylation could guarantee the stability of gelatin emulsion [4]. The advancement of the functional properties of glycosylated gelatin might be closely related to the addition of polysaccharide and glycosylation methods. Nowadays, ultrasonic-assisted glycosylation (UG) has been used as an efficient method to increase the gelling properties of proteins [11]. However, as far as we know, there have still been few basic reports on the impacts of UG on the functional properties (e.g., gelling, rheological, and emulsifying properties) and structural characteristics of FG.

Glucose (GLU), is a kind of reducing sugar that consists of a carbohydrate compound containing six C- atoms and a CH_3_CHO. GLU is referred to as aldohexose. A study proved that glycosylated protein with glucose showed high emulsifying properties [12]. In addition, some studies have shown that UG could change the gelling, emulsifying, and structural properties of protein [11,13]. However, the synergetic effects of ultrasonic and glycosylation (UG) with GLU on the functional (emulsifying, gelling, and rheological properties) and structural properties of FG are still unclear.

Thus, the goal of this paper was to modify FG using UG with GLU through the measurements of the glycosylation degree (DG), gel color, gel strength, texture, and rheology. Moreover, structural analysis (fluorescence, FTIR, and LF-NMR analysis) was also performed to evaluate the modification mechanism.

## 2. Results and Discussion

### 2.1. Glycosylation Degree (DG) Analysis

The DG is closely related to the functional properties of glycosylated protein [11]. A glycosylation reaction depletes the free amino acids (FAAs) of gelatin and the carbonyl group of glucose; thus, the measurements of the FAA contents could reflect the DG. As shown in Table 1, the FAAs decreased with increases in the ultrasound power and duration, indicating that UG successfully facilitated the ε-amino group of FG to graft with the reduced-terminal C=O of GLU [14]. This might be due to the fact that UG caused the gelatin to unfold, causing the Maillard reaction to easily occur and making the gelatin more reactive [15]. Thus, the higher the power and the longer the reaction time of the UG, the more FG-GLU conjugates were generated.

### 2.2. Functional Properties Analysis

#### 2.2.1. Emulsifying Properties

The emulsifying properties are some of the most important functional properties to evaluate the quality of gelatin, which includes the emulsifying ability index (EAI) and the emulsifying stability index (ESI) [16]. Table 1 shows that all the treated samples had higher EAI values compared to those of the unmodified FG, and UGFG3 had the highest EAI and ESI. This indicates that proper UG treatment significantly increased the emulsifying properties of gelatin. This could be due to the fact that the UG treatment caused more hydrophilic parts of the FG–GLU conjugates to become highly oriented to the water phase and more hydrophobic protein moieties to become firmly attached to the oil phase of the droplet surface, which provided a steric stabilizing layer to inhibit the coalescence of the emulsion droplets [17]. The surface hydrophobicity (H_0_) can be used to evaluate the degree of exposure of the hydrophobic groups in proteins. Herein, the UG treatment significantly decreased the H_0_ of all glycosylated gelatin samples, and UGFG4 had the lowest value. This suggests that the UG treatment caused a large number of GLUs containing hydrophilic –OH to be incorporated into the gelatin molecules, increasing the hydrophilicity of the proteins [15]. The larger number of formed FG–GLU conjugates showed higher steric hindrance around proteins, leading to a decrease in the H_0_.

#### 2.2.2. Foaming Properties

As shown in Table 1, the foaming capacity (FC) of the gelatin increased with increases in the ultrasonic power and reaction time, and UGFG4 had the highest FC value, indicating that the UG treatment improved the FA of gelatin prominently. UG might change the conformation structure of gelatin molecules and make gelatin molecules quickly adsorb onto the air–water interface, along with the formation of an elastic mucous membrane, resulting in improvements in the FA [18]. Moreover, the formation of FG–GLU conjugates enhanced the viscosity of a gelatin solution [19], which had a positive influence on the FA. Meanwhile, the UG treatment significantly decreased the FS of the gelatin solution and UGFG3 had the lowest value. This was attributed to the introduction of excessive -OH in the gelatin molecules, enhancing the electrostatic attraction and the interfacial tension [18], leading to a decrease in the FS.

#### 2.2.3. Gel Strength

The gel strength is one of the important indexes to evaluate the commercial value of gelatin [20]. Table 1 shows that the original gelatin possessed the highest gel strength value compared to those of the FG-GLU mixture and FG-GLU conjugate groups (except for UGFG3), and FG-GLU had the lowest value. This indicates that the addition of GLU and the UG treatment with GLU decreased the gelling properties of the gelatin to a certain extent. Theoretically, FG could interact with GLU to form a stable complex and strengthen the gel network of gelatin gels [5], but this study showed an adverse result. According to the results of Zhang et al. [21], there are three complexes: the FG-FG complex, the FG-GLU complex, and the GLU-GLU complex. The FG-FG and FG-GLU complexes could strengthen the gel networks of gelatin gels, while the GLU-GLU could not form a gel network, which contributed to the decrease in the gel strength of gelatin gels. Compared to FG-GLU, all the UG-modified gelatin possessed higher gel strength values. This indicates that the formed FG-GLU conjugates could overcome the phenomenon of GLU weakening gelatin gels [14]. This is because the glycosylation reaction reduced the H_0_ of the proteins and led to the hydrophilic groups becoming embedded in the interior of the protein molecules, contributing to the formation of hydrogen bonds among the gelatin gels.

### 2.3. Rheological Properties

#### 2.3.1. Gelatin Kinetics Analysis

**Figure 1:**
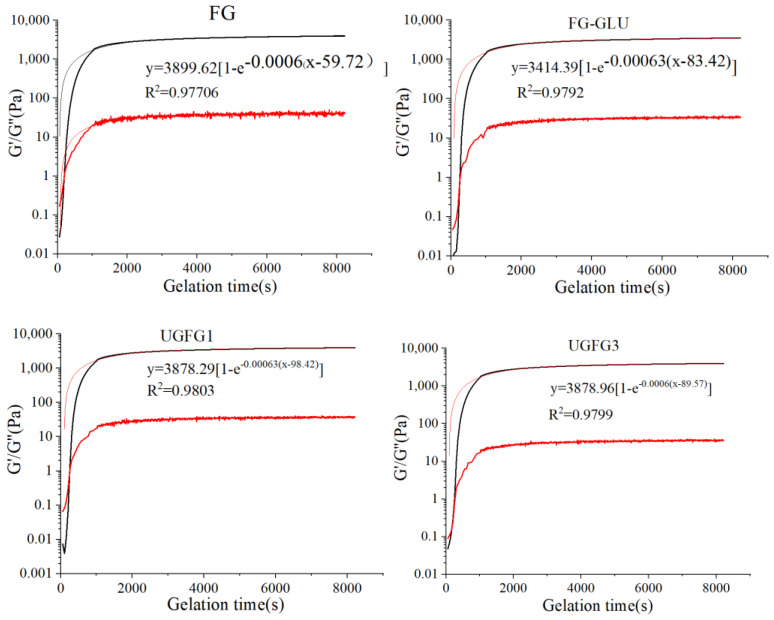
UG treatment influence on the gelation kinetics of gelatin gels. The black line represents the storage modulus (G′), the red line is the loss modulus (G″).

In order to evaluate the influence of time on the gelation behavior of UG-treated gelatin, the changes in G′ and G″ were measured to evaluate the gelation behaviors. As shown in Figure 1, at the first 1000 s, the G′ of all gelatin systems increased at a faster rate and then continued to increase at a slower rate. During the gelling process, when G′ > G″, the colloid forms. The gelatin gelation process, which involves the gelatin molecule changing from an irregular coil to a three-strand spiral, is mainly affected by the pH, gelatin concentration, temperature, and salt [1,4]. Once the colloidal network structure was formed, along with further gelation procedure, the G’ continued to increase at a slower rate, indicating the continued generation of a three-strand helix, and the gel network structure was further enhanced [22]. In this study, the original gelatin gel had a slightly higher G′_∞_ than the others, indicating that the formation of the FG-GLU complex coacervates and conjugates decreased the triple-helix contents in all treated gelatin gels [4]. Nevertheless, the constant gelation rate (k) of all gelatin gels forming curves had non-significant changes, and the modification methods obviously increased the t_g_, especially for UGFG1 and UGFG3 (Table 2). The introduction of GLU may have increased the electrostatic repulsion in the composite system, which reduced the fluidity of the FG molecules [14]. Moreover, the formation of FG-GLU conjugates may have decreased the hydrogen bonds in the gelatin, disturbing the formation of the gel.

#### 2.3.2. Flow Behaviors

**Figure 2:**
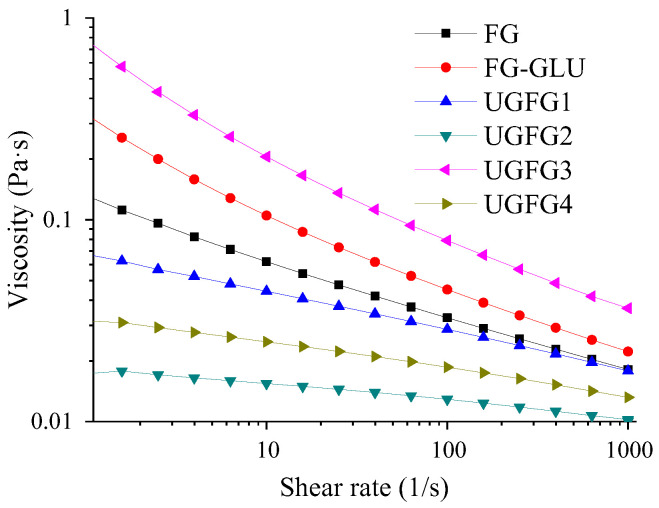
UG treatment influence on the apparent viscosity of the gelatin solution.

Figure 2 shows that the apparent viscosity of the samples decreased with the shear rate, demonstrating that the unmodified and modified gelatin solutions still had typical pseudoplastic behavior [20]. FG-GLU had a higher viscosity than that of the original FG; this might be because the FG interacted with the GLU to form a soluble complex. In theory, Maillard reaction products (MRPs) have higher molecular weights, which could contribute to the increased viscosity of the gelatin. Meanwhile, all the UG-treated samples had a lower viscosity (except UGFG3) than those of the untreated one. This may be attributed to the attachment of the GLU groups to the gelatin molecules, which changed the surface charges of the gelatin molecules. This is similar to the results of Zhao et al. [4], who reported that glycosylation with gum arabic decreased the viscosity of FG. This difference might be closely related to the treatment conditions and sugar.

### 2.4. Structural Analysis

#### 2.4.1. Fluorescence Analysis

**Figure 3:**
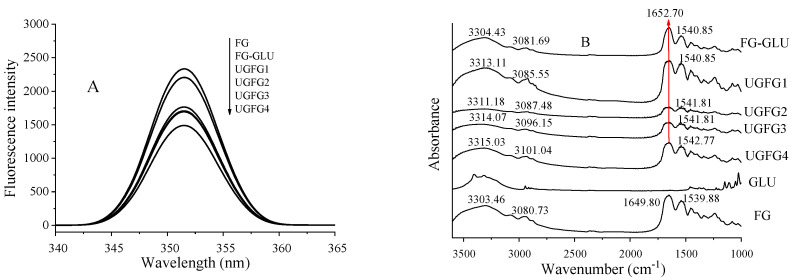
UG treatment influence on the fluorescence intensity (**A**) and FTIR (**B**) of samples.

Changes in the fluorescence can be used to evaluate the tertiary structure of proteins because the fluorescent amino acids (tyrosine and phenylalanine) in the gelatin molecules are highly sensitive to intrinsic fluorescence [23]. As shown in Figure 3A, compared to the control group, the fluorescence intensities of all modified gelatin decreased, indicating that modification could bury the tyrosine intensity [2]. The decrease in the fluorescence intensity of the UG-treated gelatin may have been due to the stretched protein molecules and the aromatic amino acid groups being exposed to a strong polar aqueous solution during the UG process. Obviously, with the increase in the ultrasonic power and processing time, the fluorescence intensity also gradually decreased, indicating that high-frequency long-term ultrasonic treatment was more conducive to protein molecule stretching.

#### 2.4.2. FTIR Analysis

FTIR analysis can be used to evaluate the characteristics of the secondary structure of a protein. Normally, gelatin has three typical absorption bands: amide I (1600–1700 cm^−1^, C-O stretching), amide II (1500–1550 cm^−1^, N-H deformation), and amide III (1200–1300 cm^−1^, C-N stretching and N-H deformation) [9]. As shown in Figure 3B, the amide I of the UG-treated gelatin shifted from 1649.80 to 1652.70 cm^−1^. This indicates that the UG treatment caused the formation of a Schiff base (with a C=N structure) [14]. The amide II of the gelatin moved from 539.88 cm^−1^ to a range of 1540.85–1542.77 cm^−1^ in all modified gelatin samples, suggesting that the N–H participated in the glycosylation reaction, along with the changes in the secondary structure of the gelatin through the reduction of the α-helix contents whilst increasing the β-turn [4,21].

Amide B corresponds to the asymmetric stretching vibrations of =C-H and NH^+^_3_ [9]. Herein, the amide B wavelength numbers increased with increases in the ultrasound power and duration, and UGFG4 had the highest values. This was due to the N–H stretching vibration caused by the introduction of GLU molecules. Amide A is closely related to the stretching vibrations of the N-H group. The UG-treated gelatin had higher amide A values than those of the original FG and FG-GLU, and the amide A slightly increased with increases in the ultrasound power and duration. This indicates that inter-molecular hydrogen bonds were formed between the O-H groups in the GLU and the N-H groups of FG [18]. This may have been caused by the introduction of a large number of GLU chains containing hydrophilic –OH during the glycosylation reaction procedure [14]. This might explain why proper UG treatment could increase the gel properties of gelatin gels.

#### 2.4.3. LF-NMR Analysis

**Figure 4:**
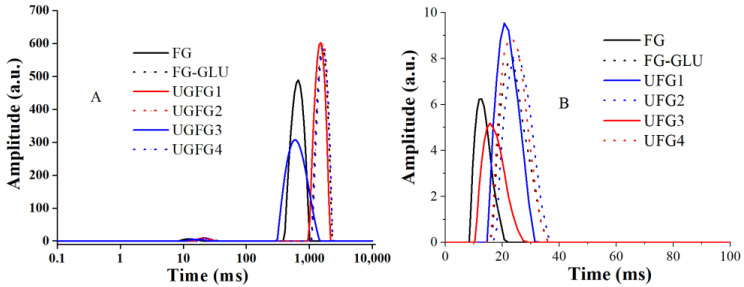
UG treatment influence on the relaxation time distribution ((**A**): 0–10,000 ms; (**B**): 0–100 ms) in the gelatin gels.

LF-NMR can be used to analyze the degree of freedom, fluidity, and distribution characteristics of water in a food system [24]. The relaxation time (T2) consists of three components, namely bound water (T21), immobilized water (T22), and free water (T23) [14]. Two water fractions, T21 (0–10 ms) and T23 (>600 ms), were identified (Figure 4). The T21 values of the original FG, FG-GLU, UGFG1, UGFG2, UGFG3, and UGFG4 were 12.75 ms, 25.53 ms, 20.73 ms, 25.53 ms, 15.70 ms, and 23.82 ms, respectively. This suggests that the modification methods decreased more bound water in the gels [22]. Moreover, compared to the FG-GLU, the UG modification could restrict more bound water contents in the gels. Thus, the UG treatment-produced MRPs could be assigned to the tightly bound water molecules, which directly interacted with the groups of proteins. The T23 values of the original FG, FG-GLU, UGFG1, UGFG2, UGFG3, and UGFG4 were 666.99 ms, 1644.68 ms, 1534.37 ms, 1644.68 ms, 580.52 ms, and 1644.68 ms, respectively. This indicates that the modification could not decrease the free water contents in the gelatin gels, except in UGFG3. Thus, proper UG treatment could constrain more water molecules, which is also consistent with the gel property results.

## 3. Conclusions

The results of this study show that high ultrasound power and a long reaction time of UG significantly decreased the FAA contents of gelatin and the formation of more MRPs. Although the UG treatment could significantly increase the EAI (76.84 ± 0.87–97.42 ± 0.86) and FC (65.83 ± 9.65–81.67 ± 4.71) of FG, it decreased the gel strength of the gelatin gels (except in UGFG3). The gelation kinetics analysis showed that the addition of GLU and UG treatment both prolonged the gelation time (83.42–98.42 s) by the restraint of triple-helix formation. The structural analysis indicated that the production of MRPs covered the fluorescence site of amino acids, changing the secondary structure of the gelatin through the reduction of the ɑ-helix contents whilst increasing the β-turn. Moreover, compared to the FG-GLU, the UG treatment accelerated the formation of inter-molecular hydrogen bonds, which restricted higher bound water contents in the gels.

## 4. Materials and Methods

### 4.1. Materials

Fish gelatin (FG, type A, protein content of 88.73 ± 0.62%) was bought from Yuanye (Shanghai, China). Glucose (GLU, D-(+)-dextrose purity of ≥ 99.5%), 1-anilinonaphthalene-8-sulfonic acid (ANS), and sodium phosphate buffer (PBS) were bought from Solebao Biotechnology Co., Ltd. (Shanghai, China). All other chemicals were pure analytical reagents.

### 4.2. Modification of FG

Proper FG powder was dissolved in ultrapure water to prepare the FG solution (5%, *w*/*v*) at 40 °C with constant stirring. Glucose was added to the FG solution at a ratio of 10:1 to prepare the FG-GLU mixture solution. The pH of the FG-GLU solution was adjusted to 9.0 using 1M NaOH. The FG-GLU solution was treated with different levels of ultrasound power (100 W, 200 W) and durations (0.5 h, 1 h). After the ultrasound treatment, the pH of the sample solution was adjusted to 6.5 using 1M HCl. The FG-GLU mixtures treated with 100 W for 0.5 h and 1 h were named UGFG1 and UGFG2, respectively, and the samples treated with 200 W for 0.5 h and 1 h were named UGFG3 and UGFG4, respectively. The non-treated fish gelatin–glucose solution was named FG-GLU. The pure FG solution was regarded as the control.

### 4.3. Degree of Grafting (DG) Analysis

The degree of grafting of all gelatin solutions was determined by measuring the free amino acid (FAA) contents of all gelatin samples using the O-phthalaldehyde (OPA) method [14]. Glycosylated gelatin solutions (0.2 mL) were mixed with 4 mL of freshly prepared OPA reagent, and then the mixture was kept at 35 °C for 2 min. The absorbance of the mixture was measured with a U-2910 UV-Vis spectrophotometer (Hitachi, Ltd., Tokyo, Japan) at 340 nm. Ultrapure water was used as the control group. The FAA content was calculated using a Lys standard curve. The DG was calculated using the following equation:

(1) $$DG=\frac{A_{0}-A_{t}}{A}\times 100\%$$

where *A*_0_ refers to the FAA contents of the FG-GLU mixture; *A_t_* refers to the FAA contents of the FG- GLU conjugates; A refers to the FAA contents of the FG.

### 4.4. Functional Properties Analysis

#### 4.4.1. Emulsifying Properties

The emulsifying properties were measured according to the reports of Bi et al. [15]. The gelatin solution (30 mL 1.0 mg/mL) was mixed with 10 mL of soybean oil, and then homogenized at a speed of 10,000 rpm for 2 min using a dispersion machine (Ultra-Turrax T25, IKA, Germany). After dispersion, 50 μL of the emulsion was taken immediately from the bottom of the emulsion and then immediately mixed well with 5 mL of 0.1% sodium dodecyl sulfate (SDS) solution. An absorbent value of 500 nm (Abs_500_) was determined using a UV-Vis spectrophotometer. After 10 min, 50 μL of the emulsion was taken from the bottom of the emulsion and mixed well with 5mL of 0.1% SDS solution again. The Abs_500_ values at 0 and 10 min were named *A*_0_ and *A*_10_, respectively. The emulsifying ability index (EAI) and emulsifying stability index (ESI) were computed using the following formulas:

(2) EAI(m2g)=2×2.303×A0×DFc×Φ×I

(3) $$ESI(\min)=\frac{A_{0}}{A_{0}-A_{10}}\times \Delta t$$

where *A*_0_ and *A*_10_ refer to the Abs_500_ of the emulsion at 0 and 10 min, respectively; *DF* is the dilution factor (×50); *c* means the weight of protein per volume (1000 g/m^3^); Φ is the oil portion of the emulsion (0.25); *I* means the path length of the cuvette (1 cm); Δ*t* was 10 min.

#### 4.4.2. Foaming Properties

The gelatin solution (20 mL, 5 mg/mL) was dispersed at a speed of 9500 rpm for 2 min by a dispersion machine (Ultra-Turrax T25, IKA, Germany), and then the dispersed solution was poured into a 50 mL cylinder immediately. The volume of foam was recorded as *V*_1_, and after 30 min, the volume of the solution was recorded as *V*_2_ [16]. The *FC* and *FS* were calculated using the following formulas:

(4) $$FC(\%)=\frac{V-20_{1}}{20}\times 100$$

(5) $$FS(\%)=\frac{V_{2}}{V_{1}-20}\times 100$$

#### 4.4.3. Surface Hydrophobicity(H_0_) Analysis

The H_0_ of each sample was measured using ANS as a probe [16]. Various gelatin solutions were obtained using 0.01 mM of PBS (pH 7.2). Then, 10 mL of solution was mixed with 0.1 mL of ANS and held for 15 min in the darkness. The fluorescence intensity (FI) of each mixture was obtained using a fluorescence spectrophotometer (F4700, Hitachi Co., Tokyo, Japan). The emission wavelength and excitation wavelength were 485 nm and 374 nm, respectively. Finally, the obtained initial slope was analyzed using a linear regression analysis of the FI versus the gelatin concentration, and this value was defined as the H_0_.

#### 4.4.4. Gel Strength Analysis

The gel strengths of the gelatin samples were measured using the Texture Analyzer (Stable Micro System, Surrey, UK), which was equipped with a cylindrical aluminum probe (P 0.5R). The gelatin solution was poured into a 25 mL glass beaker and incubated at 10 °C in a refrigerator for 16–18 h. When the penetration distance of the probe reached 4 mm with 1 mm/s, the gel strength was recorded. This process was performed in triplicate for each sample.

### 4.5. Rheological Properties Analysis

#### 4.5.1. Gelation Kinetics

The gel formation dynamics of the gelatin were evaluated based on the report of Zhao et al. [4]. The gelatin solutions were cooled from 25 °C to 4 °C at a speed of 1 °C/min. The frequency was 0.5 Hz, and the strain was 0.5%. After that, the formed gels were held at 4 °C for 2 h. The storage module values (*G*′) and lost module values (*G*″) were recorded for a fixed period of time. The relationship between the *G*″ and time was applied to the first-order kinetic energy model.

(6) $$G\prime (t)={G\prime }_{\infty }[1-e^{-k(t-t_{g})}]$$

where *G*′_∞_ is the estimate of the auto-limited modulus (Pa); *t* is the time (s); *t_g_* is the duration of gelation (s); *k* is the constant gelation rate.

#### 4.5.2. Flow Behavior

The flow behaviors of all gelatin solutions were evaluated by measuring the apparent viscosity using a rheometer with 60 mm of a stainless steel parallel plate. The shear rate of each sample ranged from 0.01 to 100 s^−1^. The gap value was 1000 μm [25].

### 4.6. Structural Analysis

#### 4.6.1. Fluorescence Analysis

The sample solutions (0.1 mg/mL) were placed into quartz cuvettes for endogenous fluorescence measurements using a fluorescence spectrophotometer (F4700, Hitachi Co., Tokyo, Japan). The excitation wavelength was 280 nm. The scanning wavelength of each sample ranged from 290 to 490 nm with 1200 nm/min. The slit width was 5.0 nm [26].

#### 4.6.2. Fourier Transform Infrared (FTIR) Analysis

The gelatin samples were ground with KBr and then pressed into thin slices. The FTIR spectra of each sample were determined using a Spectrum One FTIR spectrometer (PerkinElmer, Waltham, MA, USA). The FTIR spectra obtained had a wavelength range of 4000–500 cm^−1^. The resolution was 4 cm^−1^, and the scan speed was 64 scans [26].

#### 4.6.3. Low-Field Nuclear Magnetic Resonance (LF-NMR)

The low-frequency NMR of the gelatin gels was determined using a MesoMR23-060H-I Analyst NMR analyzer (Suzhou Tainiu Testing Service Co., Ltd., Suzhou, China) at 32 °C. The gelatin (2 g, 5%, *w*/*v*) was placed in a glass tube. The continuous scan time was set to 10 s, the echo time was 0.5 ms, and the echo frequency was 8000. The number of scans was 32 and the pulse spacing (s) ranged from 90 to 180 within 250 s [22].

### 4.7. Statistical Analysis

Each experiment was measured in triplicate and the reported results are presented as the mean values ± standard deviation. The data were analyzed using SPSS statistics 23.0 (SPSS Inc., Chicago, IL, USA), and a *p* < 0.05 was considered significant.

## Figures and Tables

**Table 1:** Ultrasound-assisted glycosylation (UG) influenced the glycosylation degree, emulsifying properties, foaming properties, surface hydrophobicity (H_0_), and gel strength of FG.

Samples	FAA (mg/mL)	DG (%)	EAI (m^2^/g)	ESI (%)	FC (%)	FS (%)	H_0_	Gel Strength (g)
FG	0.152 ± 0.004 ^f^	-	73.62 ± 1.30 ^a^	92.42 ± 8.92 ^de^	60.83 ± 4.25 ^a^	240.97 ± 21.42 ^bc^	303.23 ± 10.70 ^cd^	300.14 ± 32.96 ^de^
FG-GLU	0.147 ± 0.001 ^e^	-	79.07 ± 0.27 ^c^	51.41 ± 9.01 ^a^	65.00 ± 7.36 ^ab^	230.17 ± 18.99 ^b^	289.13 ± 9.74 ^c^	252.05 ± 7.30 ^a^
UGFG1	0.097 ± 0.001 ^d^	33.14 ± 0.85 ^a^	76.84 ± 0.87 ^b^	83.21 ± 8.79 ^c^	65.83 ± 9.65 ^ab^	203.65 ± 24.92 ^ab^	263.17 ± 8.63 ^ab^	280.74 ± 6.66 ^d^
UGFG2	0.082 ± 0.005 ^c^	45.37 ± 0.24 ^b^	86.78 ± 0.76 ^d^	72.76 ± 1.74 ^b^	79.83 ± 5.22 ^bc^	200.79 ± 12.49 ^ab^	258.50 ± 9.32 ^a^	270.34 ± 2.22 ^c^
UGFG3	0.075 ± 0.01 ^b^	47.42 ± 1.06 ^c^	97.42 ± 0.86 ^f^	98.84 ± 2.50 ^e^	70.17 ± 5.89 ^b^	195.24 ± 14.97 ^a^	270.30 ± 5.88 ^b^	309.43 ± 0.41 ^e^
UGFG4	0.06 ± 0.001 ^a^	57.39 ± 0.49 ^d^	94.77 ± 1.53 ^e^	91.17 ± 9.10 ^de^	81.67 ± 4.71 ^c^	199.88 ± 16.46 ^a^	252.03 ± 6.94 ^a^	258.71 ± 0.18 ^ab^

FG: fish gelatin; FG-GLU: fish gelatin–glucose; UGFG1, UGFG2: FG-GLU was treated by 100 W of ultrasound-assisted glycosylation for 0.5 h and 1 h, respectively; UGFG3, UGFG4: FG-GLU was treated by 200 W of ultrasound-assisted glycosylation for 0.5 h and 1 h, respectively. Lowercase letters (a–f) in the same column indicate significantly different levels (*p* < 0.05).

**Table 2:** Gelation kinetics analysis.

Gelling Systems	G′_∞_ (Pa)	K (1/s)	t_g_ (s)	R^2^
FG	3899.62	0.0006	59.72	0.977
FG-GLU	3414.39	0.0006	83.42	0.979
UGFG1	3878.29	0.0006	98.42	0.980
UGFG3	3878.96	0.0006	89.57	0.980

## References

[1] Yang H.J., Wang H.F., Huang M., Cao G.T., Tao F., Zhou G.H., Shen Q., Yang H.S. (2022). Repurposing fish waste into gelatin as a potential alternative for mammalian sources: A review. Compr. Rev. Food Sci. Food Saf..

[2] Huang T., Tu Z.C., Shangguan X.C., Sha X.M., Wang H., Zhang L., Bansal N. (2019). Fish gelatin modifications: A comprehensive review. Trends Food Sci. Technol..

[3] Li X., Wang L., Liu G.X., Tu Z.C. (2021). Effect of urea on glycosylation of BSA based on spectral techniques. Spectrosc. Spectr. Anal..

[4] Zhao H.Z., Kang X.Z., Zhou X.L., Tong L., Yu W.W., Zhang J.J., Yang W.G., Lou Q.M., Huang T. (2021). Glycosylation fish gelatin with gum Arabic: Functional and structural properties. LWT.

[5] Huang T., Zhao H.Z., Fang Y.Y., Lu J.P., Yang W.G., Qiao Z.H., Lou Q.M., Xu D.L., Zhang J.J. (2019). Comparison of gelling properties and flow behaviors of microbial transglutaminase (MTGase) and pectin modified fish gelatin. J. Texture Stud..

[6] Kolodzlejska I., Kaczorowski K., Piotrowska B., Sadowska M. (2004). Modification of the properties of gelatin from skins of Baltic cod (*Gadus morhua*) with transglutaminase. Food Chem..

[7] Kaewruang P., Benjakul S., Prodpran T. (2014). Characteristics and gelling property of phosphorylated gelatin from the skin of unicorn leatherjacket. Food Chem..

[8] Bhat R., Karim A.A. (2009). Ultraviolet irradiation improves gel strength of fish gelatin. Food Chem..

[9] Tu Z.C., Huang T., Wang H., Sha X.M., Shi Y., Huang X.Q., Man Z.Z., Li D.J. (2015). Physico-chemical properties of gelatin from bighead carp (*Hypophthalmichthys nobilis*) scales by ultrasound-assisted extraction. J. Food Sci. Technol..

[10] Da Silva R.S.G., Pinto L.A.A. (2012). Physical cross-linkers: Alternatives to improve the mechanical properties of fish gelatin. Food Eng. Rev..

[11] Lin D.R., Zhang Q.T., Xiao L.J., Huang Y.C., Yang Z.F., Wu Z.J., Tu Z.C., Qin W., Chen H., Wu D.T. (2021). Effects of ultrasound on functional properties, structure and glycation properties of proteins: A review. Crit. Rev. Food Sci. Nutr..

[12] Huang X.Q., Tu Z.C., Wang H., Zhang Q.T., Hu Y.M., Zhang L., Niu P.P., Shi Y., Xiao H. (2013). Glycation promoted by dynamic high pressure microfluidisation pretreatment revealed by high resolution mass spectrometry. Food Chem..

[13] Liu G.X., Tu Z.C., Yang W.H., Wang H., Zhang L., Ma D., Huang T., Liu J., Li X. (2018). Investigation into allergenicity reduction and glycation sites of glycated β-lactoglobulin with ultrasound pretreatment by high-resolution mass spectrometry. Food Chem..

[14] Sheng L., Liu Q., Dong W.Y., Cai Z.X. (2022). Effect of high intensity ultrasound assisted glycosylation on the gel properties of ovalbumin: Texture, rheology, water state and microstructure. Food Chem..

[15] Bi W.W., Ge W., Li X.D., Du L.L., Zhao G.X., Wang H.X., Qu X.W. (2017). Effects of ultrasonic pretreatment and glycosylation on functional properties of casein grafted with glucose. J. Food Process. Preserv..

[16] Sha X.M., Hu Z.Z., Tu Z.C., Zhang L.Z., Duan D.L., Huang T., Wang H., Zhang L., Li X., Xiao H. (2018). Influence of dynamic high pressure microfluidization on functional properties and structure of gelatin from bighead carp (*Hypophthalmichthys nobilis*) scale. J. Food Process. Preserv..

[17] Zhang T., Sun R., Ding M.Z., Li L., Tao N.P., Wang X.C., Zhong J. (2020). Commercial cold-water fish skin gelatin and bovine bone gelatin: Structural, functional, and emulsion stability differences. LWT.

[18] Li S.G., Zhang S., Liu Y., Fu X., Xiang X.L., Gao S.H. (2022). Effects of ultrasound-assisted glycosylation on the interface and foaming characteristics of ovotransferrin. Ultrason. Sonochem..

[19] Liu L., Wang Q., Wu Y.Y., Wang G.Z., Geng F., Song H.B., Luo P., Huang Q. (2022). Effect of ball milling-assisted glycosylation modification on the structure and foaming property of egg white protein. J. Food Sci..

[20] Lu J.P., Fang Q., Ma N., Yang W.G., Zhang L.Y., Huang T. (2022). Gelation behaviour of fish skin gelatin in the presence of methanol-water and ethanol-water solvent system. Int. J. Food Sci. Technol..

[21] Zhang Y., Xu J., Zhang T., Tao L., Nie Y., Wang X., Zhong J. (2022). Effect of carbon numbers and structures of monosaccharides on the glycosylation and emulsion stabilization ability of gelatin. Food Chem..

[22] Cen S.J., Zhang L.Y., Liu L.W., Lou Q.M., Wang C.C., Huang T. (2022). Phosphorylation modification on functional and structural properties of fish gelatin: The effects of phosphate contents. Food Chem..

[23] Zhang H., Liu Z., Zhang J.T., Zhang L., Wang S., Wang L., Chen J., Zou C.H., Hu J.D. (2021). Identification of edible gelatin origins by data fusion of NIRS, fluorescence spectroscopy, and LIBS. Food Anal. Meth..

[24] Hansen M.R., Blennow A., Farhat I., Norgaard L., Pedersen S., Engelsen S.B. (2009). Comparative NMR relaxometry of gels of amylomaltase-modified starch and gelatin. Food Hydrocolloid..

[25] Kang X.Z., Guo W.W., Ding K.Y., Zhan S.N., Lou Q.M., Huang T. (2022). Microwave processing technology influences the functional and structural properties of fish gelatin. J. Texture Stud..

[26] Fang Q., Ma N., Ding K.Y., Zhan S.N., Lou Q.M., Huang T. (2021). Interaction between Negatively Charged Fish Gelatin and Cyclodextrin in Aqueous Solution: Characteristics and Formation Mechanism. Gels.

